# Amorphous Ge-Bi-Se Thin Films: A Mass Spectrometric Study

**DOI:** 10.1038/s41598-019-55773-9

**Published:** 2019-12-16

**Authors:** Ravi Mawale, Govinda Mandal, Marek Bouška, Jan Gutwirth, Pankaj Lochan Bora, Virginie Nazabal, Josef Havel, Petr Němec

**Affiliations:** 1 Department of Graphic Arts and Photophysics, Faculty of Chemical Technology, University of Pardubice, Studentská 573, 53210 Pardubice, Czech Republic; 2 Department of Chemistry, Faculty of Science, Masaryk University, Kamenice 5/A14, 625 00 Brno, Czech Republic; 3 Institut des Sciences Chimiques de Rennes, UMR-CNRS 6226, Equipe Verres et Céramiques, Université de Rennes 1, 35042 Rennes, France

**Keywords:** Mass spectrometry, Surfaces, interfaces and thin films

## Abstract

The Ge-Bi-Se thin films of varied compositions (Ge content 0–32.1 at. %, Bi content 0–45.7 at. %, Se content 54.3–67.9 at. %) have been prepared by rf magnetron (co)-sputtering technique. The present study was undertaken in order to investigate the clusters generated during the interaction of laser pulses with Ge-Bi-Se thin films using laser ablation time-of-flight mass spectrometry. The stoichiometry of the clusters was determined in order to understand the individual species present in the plasma plume. Laser ablation of Ge-Bi-Se thin films accompanied by ionization produces about 20 positively and/or negatively charged unary, binary and ternary (Ge_*x*_^+^, Bi_*y*_^+^, Se_*z*_^+/−^, Ge_*x*_Se_*z*_^+/−^, Bi_*y*_Se_*z*_^+/−^ and Ge_*x*_Bi_*y*_Se_*z*_^−^) clusters. Furthermore, we performed the laser ablation experiments of Ge:Bi:Se elemental mixtures and the results were compared with laser ablation time-of-flight mass spectrometry analysis of thin films. Moreover, to understand the geometry of the generated clusters, we calculated structures of some selected binary and ternary clusters using density functional theory. The generated clusters and their calculated possible geometries can give important structural information, as well as help to understand the processes present in the plasma processes exploited for thin films deposition.

## Introduction

Amorphous chalcogenides have received great attention due to their unique properties and a broad range of applications. Chalcogenide compounds have emerged as multipurpose material and have been used for various applications for solid-state devices^1^. Due to their specific properties, chalcogenide glasses and their amorphous thin films are applied in photonics^2^, solar cells^3^, phase change memories^4,5^, optical fibers^6^, etc.

Among many chalcogenide glasses, selenium-based ones are significant because of their wide transparency window in the infrared spectral region, strong non-linear optical properties, high refractive index, and good mechanical, thermal and chemical characteristics^2,7–9^. For example, especially germanium selenides show good mechanical properties, such as hardness, adhesion, low internal stress, and water resistance^10,11^. In fact, Ge-Se glasses are thoroughly studied from the point of view of glass-forming region, their structural features, and physical properties^11,12^. Physical properties of binary or ternary chalcogenide glasses based on the Ge-Se system can be further adjusted by adding other elements. Specifically, the incorporation of Bi to the chalcogenide Ge-Se-based glasses and thin films leads to a very rare effect, namely n-type conductivity due to carrier reversal change, while the addition of other elements of the same group (As, Sb) does not^13–16^. The observed n-type conductivity may be related to the increased metallic properties of bismuth as compared to As and Sb; hence an addition of bismuth may control, in an effective way, electrical/optical properties of Ge-Se-based glasses and thin films. As a consequence, amorphous Ge-Bi-Se chalcogenide thin films are investigated to study their optical and electrical properties^17–19^, structure^17,20,21^, etc.

Various deposition techniques are used for the fabrication of thin films from chalcogenide glasses. These are, for example, pulsed laser deposition, thermal evaporation, chemical vapor deposition, radio-frequency magnetron sputtering, etc^22^. Of special interest is rf magnetron co-sputtering, which may produce thin films with different stoichiometries by varying only the electrical power applied to the individual sputtering targets. Indeed, co-sputtering is a cost-effective process for the investigation of compositional dependencies of thin films’ characteristics. One of co-sputtering’s key features is that it enables the fabrication of amorphous thin films with compositions outside the glass-forming region of corresponding bulk counterparts^23,24^.

As mentioned above, different characterization techniques have been employed to examine Ge-Bi-Se glasses and amorphous thin films in order to understand their characteristics. It has been shown that irradiation of amorphous chalcogenides – in their bulk as well as thin film forms – with a pulsed laser produces many clusters via laser ablation processes, as confirmed through time-of-flight mass spectrometry. This technique can be used to elucidate the structural fragments present in the plasma generated during the interaction of high energy laser pulses with chalcogenide glasses^25–27^. However, mass spectrometric study of Ge-Bi-Se materials has not yet been reported, and therefore it is carried out here.

The aim of this work is to investigate Ge-Bi-Se amorphous chalcogenide thin films fabricated via rf magnetron co-sputtering. The fabricated films were characterized in detail using laser ablation time-of-flight mass spectrometry (LA TOFMS) analysis. The stoichiometry of the species generated from parafilm coated and non-coated amorphous Ge-Bi-Se thin films was determined and the results were discussed. Further, we also compared the clusters generated from Ge-Bi-Se layers with those originating in mixtures of Ge, Bi, and Se elements. Finally, for selected binary and ternary species, their structure was calculated using density functional theory (DFT).

## Results and Discussion

The thin films of different compositions, labelled A-F in Table 1, prepared via magnetron co-sputtering were characterized by scanning electron microscopy; the images show that the morphology of the layers is of good quality with smooth surfaces, and marginal presence of inhomogeneities. The elemental composition of the fabricated thin films was obtained by EDS at different spots of the films and averaged (Table 1). The EDS data indicate that single cathode sputtering (at 20 W) gives thin films with a composition in line with the GeSe_2_ and Bi_2_Se_3_ targets used. In case of co-sputtering, by using rf power 15–29 and 6–20 W for GeSe_2_ and Bi_2_Se_3_ targets respectively, the co-sputtered films cover a wide compositional range: 6.1–27.6 at. % of Ge, 7.0–35.7 at. % of Bi, and 58.2–65.4 at. % of Se. XRD patterns revealed that all the deposited films (except Bi_2_Se_3_) are amorphous. The thickness of the thin films are within the range 660–1010 nm (±2 nm).

**Table 1:** Thin films identification, rf sputtering power used on both sputtering targets, and chemical composition of the co-sputtered Ge-Bi-Se thin films determined using EDS technique (±1 at. %).

Sample name	rf power (W)		Chemical composition (at. %)		
	*GeSe*_2_	*Bi*_2_*Se*_3_	*Ge*	*Bi*	*Se*
A	20	0	32.1	0	67.9
B	29	6	27.6	7.0	65.4
C	25	10	20.5	16.5	63.0
D	21	14	12.4	26.5	61.1
E	15	20	6.1	35.7	58.2
F	0	20	0	45.7	54.3

The co-sputtered Ge-Bi-Se thin films, as well as elemental mixtures of Ge, Bi, and Se, were examined via LA TOFMS in both positive and negative ion modes. During LA accompanied by ionization, the interaction of laser pulses with the materials under investigation generated many positively and negatively charged ions as well as clusters. The peaks with intensities higher than 1 mV are considered as relevant and their stoichiometries were determined by comparing experimental and theoretical isotopic patterns.

The effect of laser power on the generation of clusters was studied by systematically increasing the laser energy (90–180 a.u.). Through this procedure, the threshold energy at which ionization starts was determined for each sample in both positive and negative ion modes. For thin films, the threshold energy was identified between 120–130 a.u., while for the elemental mixtures of Ge, Bi, and Se, it is 90–100 a.u. No signals were obtained after m/z 1200, therefore, only mass spectra up to this m/z value are shown in the figures. It was noticed that the intensity of the peaks rises with increasing laser energy; however their mass resolution is decreased. Therefore, the ‘optimal’ mass spectra with sufficiently high mass resolution and the largest number of clusters were obtained for each sample in both modes of measurement. In addition, the parafilm coated thin films, as well as elemental mixtures dispersed in parafilm solution, were also examined and the results compared.

### Ge-Bi-Se thin films

#### Positive ion mode

Co-sputtered amorphous Ge-Bi-Se thin films were investigated via laser ablation TOFMS. Mass spectra with sufficient resolution and maximum number of detected species were obtained at laser energy between 140–150 a.u.

**Figure 1:**
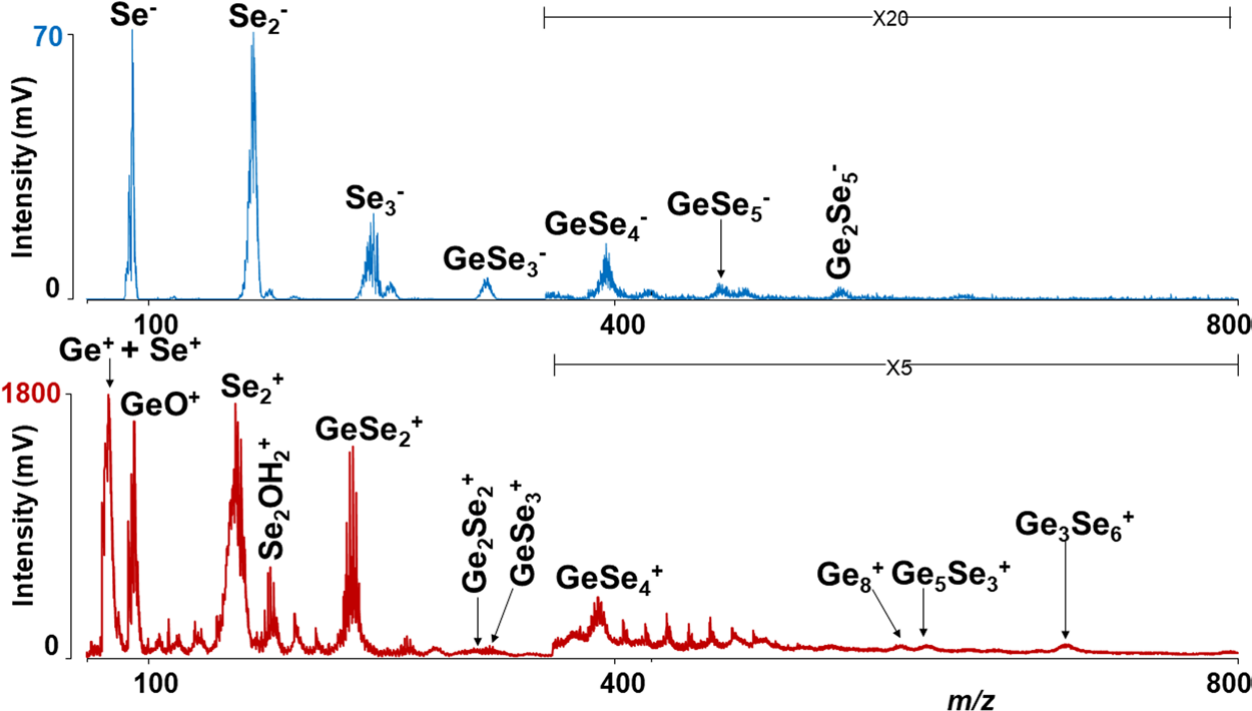
Mass spectra of sample A. Conditions: positive (red)/negative (blue) ion modes, laser energy 145 and 140 a.u. respectively.

LA of GeSe_2_ binary thin (sample A) film generated many unary and binary species (Fig. 1). The lowest mass positively charged ion obtained from the GeSe_2_ thin film is Ge^+^, which is partially overlapped with Se^+^. Several other binary Ge_x_Se_z_^+^ clusters are also detected. In addition, other unary Se_2_^+^, Ge_8_^+^ and some oxygenated species were identified. The oxygenated species might be sourced from the partial oxidation of the surface of the thin films. The results obtained from thin films A are in agreement with our previously published results from the binary Ge-Se system^27^. However, due to the somewhat lower resolution of the instrument used in this work, the stoichiometry of some peaks could not be determined. For the sample F (Bi_2_Se_3_), the lowest mass ion detected was Se^+^, and many binary Bi_y_Se_z_^+^ as well as unary Bi_y_^+^ clusters were detected, of which Bi^+^ ion has the highest intensity.

**Figure 2:**
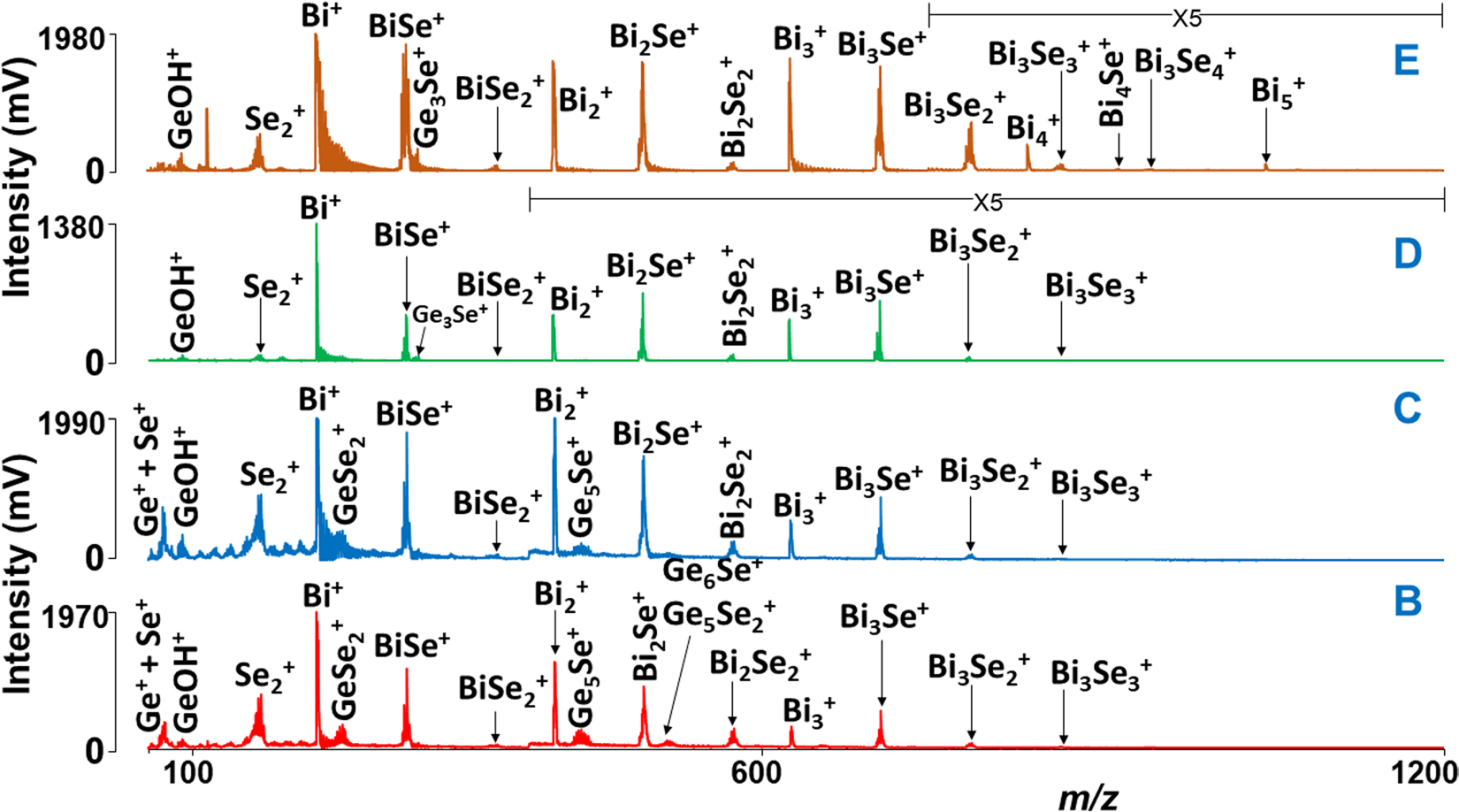
Mass spectra of Ge-Bi-Se thin films. Conditions: positive ion mode, laser energy 150 a.u.

The measured LA TOFMS data for ternary Ge-Bi-Se thin films (samples B, C, D, and E) are shown in Fig. 2. The stoichiometry of clusters obtained in all these samples is not significantly different except their intensities. The lowest mass clusters obtained in sample B and C is Ge^+^, which is overlapped with Se^+^ cation. Several series of other clusters were detected. For example, sample E shows Bi_*y*_^+^ (*y* = 1–5), Bi_*y*_Se^+^ (*y* = 1–4), Bi_3_Se_*z*_^+^ (*z* = 1–4), i.e. series of clusters with increasing number of Bi or Se atoms. The Bi^+^ ion is the highest intensity peak observed in all the samples, followed by BiSe^+^. It was observed that the Bi_*y*_^+^ or Bi_*y*_Se^+^ clusters dominate the mass spectra with their intensity and number of clusters as compared to the Ge^+^ or Ge_*x*_Se_*z*_^+^ ones. This seems to be related to the chemical composition of thin films. From ternary Ge-Bi-Se layers, sample B has lowest Bi and highest Ge content, while for sample E it is the opposite; therefore as one goes from sample B to E, the number of Ge^+^ or Ge_*x*_Se_*z*_^+^ species and their intensities are reduced. Logically, the number of Bi_*y*_^+^ or Bi_*y*_Se^+^ species and their intensities show an opposite trend. It is worth noting that no binary Ge_*x*_Bi_*y*_^+^ or ternary Ge_*x*_Bi_*y*_Se_*z*_^+^ clusters were detected in positive ion mode.

In addition, parafilm coated thin films were examined and the mass spectra were recorded. However, no significant difference in the mass spectra was observed as compared to the mass spectra of non-coated thin films; therefore, the mass spectra are not shown here. The intensity of the peaks in the mass spectra of coated thin films is lower as compared to non-coated thin films.

#### Negative ion mode

**Figure 3:**
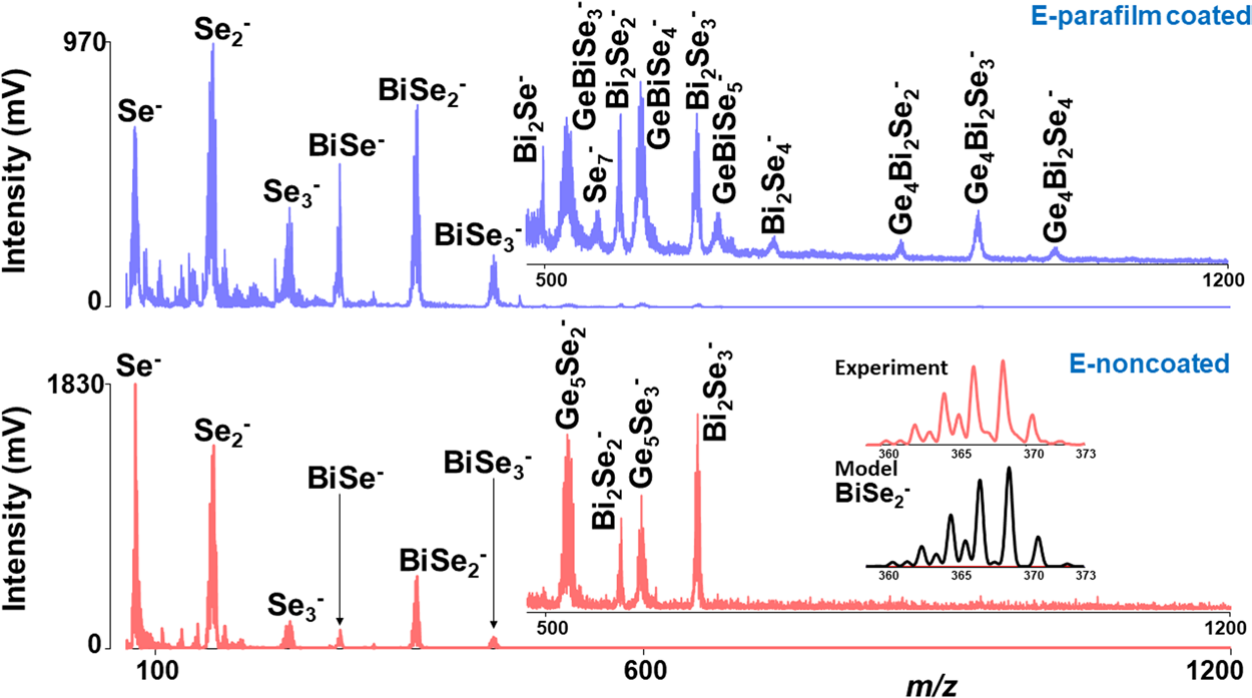
Mass spectra of parafilm coated and non-coated thin film E. Conditions: negative ion mode, laser energy 155 a.u. Part of mass spectra in the range *m/z* 490–1200 is magnified.

In negative ion mode, the threshold energy was determined to be between 120–130 a.u. for all the samples. The ‘richest’ mass spectra with sufficient mass resolution and with a maximum number of clusters were obtained at laser energy 140–155 a.u. like in positive ion mode. LA of binary GeSe_2_ thin film generated Se_*z*_^−^ (*z* = 1–3), GeSe_*z*_^−^ (*z* = 3–5), and Ge_2_Se_5_^−^ species (Fig. 1, blue spectrum). In the case of the Bi_2_Se_3_ thin films, only negatively charged Se_*z*_^−^ (*z* = 1–4) clusters were identified. When samples B, C, D, E were investigated by LA TOFMS, no significant differences between individual mass spectra were observed with respect to the number and stoichiometry of the detected species. As an example, the mass spectrum of sample E is given in Fig. 3. Series of unary Se_*z*_^−^ (*z = *1–3) and binary BiSe_*z*_^−^ (z = 1–3) species were detected. In addition, Bi_2_Se_2_^−^ and Bi_2_Se_3_^−^ and a few Ge_*x*_Se_*z*_^−^ clusters were identified. A comparison of the experimental and theoretical isotopic patterns of the selected cluster (BiSe_2_^−^), which shows their good agreement, is given in the inset of Fig. 3. It was observed that in negative ion mode, the selenium clusters are more intensive as compared to most of Bi_*y*_Se_*z*_^−^ clusters. Also, negatively charged Ge or Bi species were not detected. This might be due to the electron affinity value of selenium which is higher than both Ge and Bi (electron affinity values of Se, Ge, and Bi are 2.02, 1.23, and 0.94)^28^.

When mass spectra were recorded from parafilm coated Ge-Bi-Se thin films, new higher mass ternary clusters (GeBiSe_3_^−^, GeBiSe_4_^−^, GeBiSe_5_^−^, Ge_4_Bi_2_Se_2_^−^, Ge_4_Bi_2_Se_3_^−^, Ge_4_Bi_2_Se_4_^−^) with an increasing number of selenium atoms were detected. In addition, new species such as Bi_2_Se^−^, Se_7_^−^ and Bi_2_Se_4_^−^ were generated. The comparison of mass spectra from parafilm coated and non-coated thin films is given in Fig. 3. As in positive ion mode, it was observed that the intensity of the mass spectra peaks in negative ion mode is lower for parafilm coated thin films than for uncoated ones. This indicates that higher laser energy is required for the ionization of the ions for the coated thin films, which is also supported by the fact that higher threshold energy was observed for coated thin films.

#### Laser ablation of Ge:Bi:Se elemental mixtures

**Figure 4:**
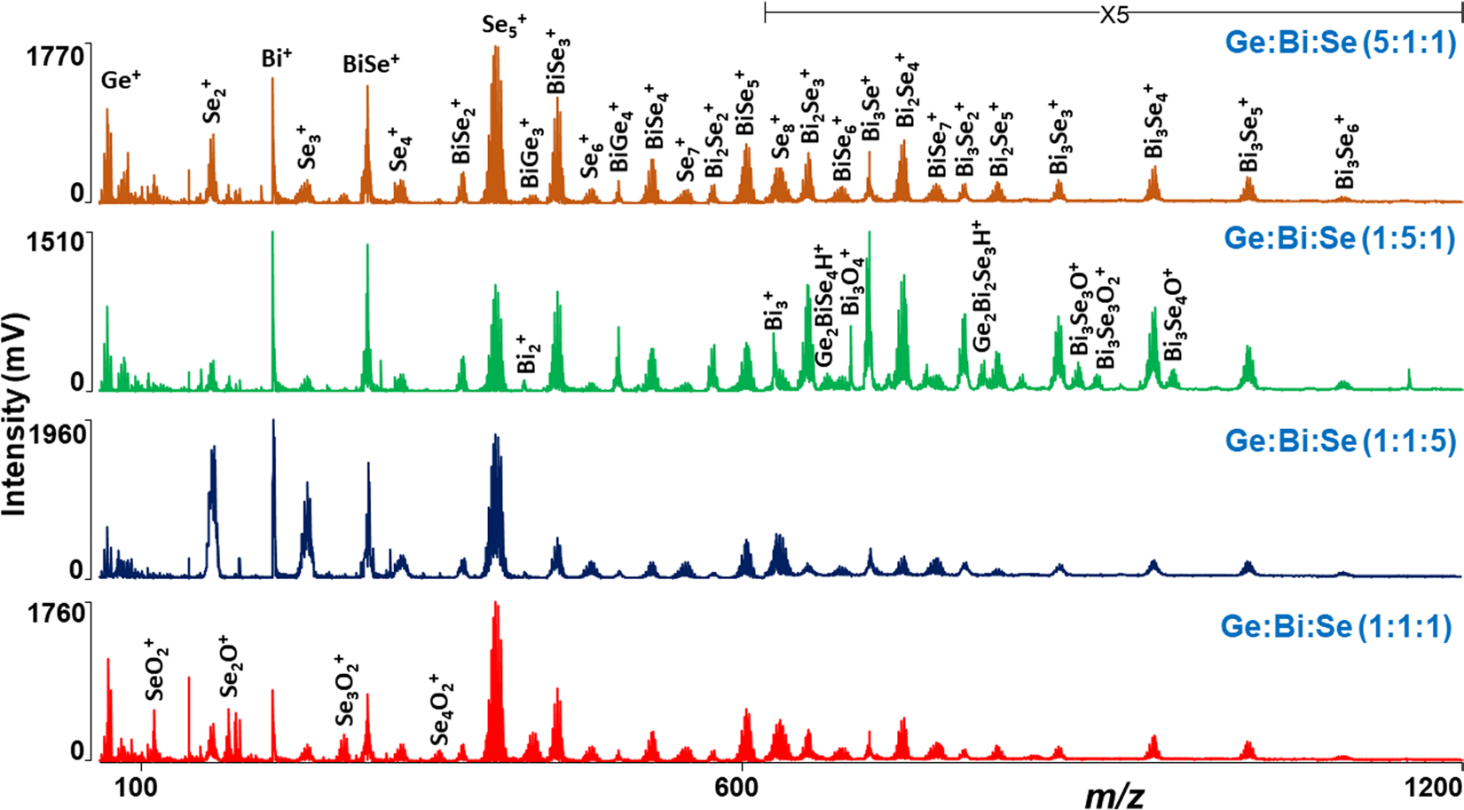
Mass spectra obtained from Ge:Bi:Se elemental mixtures. Conditions: Positive ion mode, laser energy 120 a.u. For the sake of clarity, a detailed description of most of the peaks of Ge:Bi:Se 5:1:1 precursor is given.

Later, we deal with the LA TOFMS of elemental mixtures of Ge, Bi, and Se with different molar ratios (1:1:1, 1:1:5, 1:5:1, and 5:1:1). To record the mass spectra from elemental mixtures, a similar methodology and experimental conditions as for the thin films were employed. The mass spectra were recorded at varied laser energy between 70–180 a.u.; threshold energy was determined to be between 90–100 a.u. In positive ion mode, mass spectra obtained from all the Ge:Bi:Se precursors show the formation of many unary, binary and a few ternary clusters. A comparison of mass spectra obtained from all the Ge:Bi:Se elemental mixture precursors are given in Fig. 4. Typically, unary Ge^+^, Bi^+^ ions, and Se_z_^+^ (z = 2–8) species were observed. Most of the binary clusters correspond to the Bi_y_Se_z_^+^ general formula with an increasing number of Bi and Se atoms. For example, these are BiSe_z_^+^ (z = 1–7), Bi_2_Se_z_^+^ (z = 2–5), and Bi_3_Se_z_^+^ (z = 1–6). Moreover, BiGe_3_^+^ and BiGe_4_^+^ binary species were detected. Particularly, for the LA of Ge:Bi:Se (1:1:1) the highest intensity peak corresponds to the Se_5_^+^ cluster, while several selenium oxygenated species were also identified. In the mass spectra of Ge:Bi:Se (1:1:5), where selenium is in excess, all the above-mentioned clusters were found. Logically, due to excess selenium, the peaks assigned to the selenium clusters are of higher intensity than the rest of the other peaks in the mass spectra. Nevertheless, the intensity of the peak connected with the Bi^+^ ion is the highest among all the peaks. This observation can be due to a lower value of ionization energy reported for Bi^+^ compared with Se^+^ (16.7 vs. 21.1 eV)^29^. In the case of the mass spectra recorded from the (1:5:1) precursor with an excess of bismuth, Bi_y_^+^ (y = 1–3) ion/clusters were revealed. Few ternary hydrogenated Ge_x_Bi_y_Se_z_^+^ clusters (Ge_2_BiSe_4_H^+^ and Ge_2_Bi_2_Se_3_H^+^) were also detected. LA also generated several oxygenated bismuth and bismuth selenide clusters. Among all the laser ablation generated clusters from the Ge:Bi:Se (5:1:1) precursor, Se_5_^+^ and Bi^+^ are the highest intensity peaks, even though the elemental mixture contains an excess of germanium. Surprisingly, the laser ablation synthesis generated a high number of Bi_y_Se_z_^+^ clusters rather than Ge_x_^+^ clusters. This might indicate that the ionization efficiency of germanium is lower compared to Bi and Se. The mass spectra of Ge:Bi:Se elemental mixtures dispersed in parafilm were also measured, however no significant difference in comparison with those without parafilm were observed.

**Figure 5:**
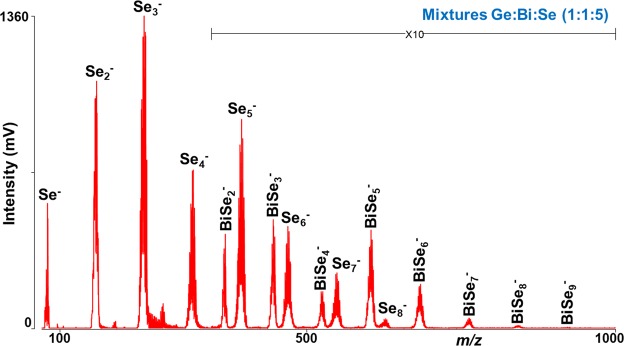
Mass spectrum of Ge:Bi:Se (1:1:5) elemental mixture. Conditions: Negative ion mode, laser energy 110 a.u.

The mass spectra recorded from Ge:Bi:Se elemental mixtures in the negative ion mode show the formation of a series of clusters such as Se_*z*_^−^ (*z* = 1–8) and BiSe_*z*_^−^ (*z* = 2–9) via laser ablation synthesis. An example of a mass spectrum of Ge:Bi:Se (1:1:5) precursor is given in Fig. 5. The Se_3_^−^ cluster represents the highest intensity peak in the mass spectra of all the precursors. However, no Ge_*x*_Se_*z*_^−^ and Ge_*x*_Bi_*y*_^−^ clusters were discovered. Especially in negative ion mode, the Se_*z*_^−^ clusters are most prominent with the highest intensity – higher than other binary Bi_*y*_Se_*z*_^−^ clusters – which indicate that selenium ionization is more efficient than Bi and Ge. The mass spectra were also measured from Ge:Bi:Se elemental mixtures dispersed in parafilm; however no significant difference in the mass spectra were observed, therefore those mass spectra are not shown.

From the results obtained via LA TOFMS of thin films and elemental mixtures, it was deduced that the threshold energy for the thin films was higher than for the elemental mixtures. Also, the number of clusters generated during the laser ablation of elemental mixtures is higher than for the thin films. An explanation for this may be that during the laser ablation of the thin films, generally, the clusters and/or ions formed may result from the original structure of solid material via its fragmentation. This process might need larger laser energy, and therefore high threshold energy was required. In contrast, in the case of the elemental mixtures, the clusters and/or ions are formed via the laser ablation synthesis of generated ion in the plasma plume, which probably needs low laser power, and hence lower threshold energy is needed.

It is worth noting that in all the recorded mass spectra from thin films and elemental mixtures, few Ge_*x*_^+/−^ clusters/ions were detected. As discussed earlier, this might be due to lower ionization of Ge when compared to Bi or Se. The results are qualitatively comparable to our previously published results, where we observed the same situation with the ionization of germanium when it is present in combination with one or more other elements^27,30^. In order to make a comparison, we have investigated elemental germanium via LA TOFMS under similar experimental conditions. The laser ablation of Ge shows the formation of positively and negatively charged Ge_*x*_^+/−^ clusters/ions with a maximum of 10 germanium atoms (Ge_10_^+/−^) (the mass spectra are not shown). However, when Ge is present in the binary (e.g. Ge-Se) or ternary (e.g. Ge-Bi-Se) systems, the pure Ge clusters are not seen (except Ge_8_^+^ and Ge^+^, cf. Figs. 1 and 2), as Ge atoms are strongly bound by Se or Bi. For example, the total number of germanium-containing clusters demonstrated in Fig. 2 is equal to 6.

Also, it is noted that the formation of Ge_*x*_Bi_*y*_^+/−^ clusters is marginal: only two (Ge_3_Bi^+^ and Ge_4_Bi^+^) cationic species were identified in mass spectra of the Ge:Bi:Se (5:1:1) elemental mixture when the content of Ge was high. To understand the process, we examined the elemental mixture (Ge:Bi, 1:1) via LA TOFMS. Surprisingly, no Ge_*x*_Bi_*y*_^+/−^ clusters were detected. However, it is difficult to judge why those species were not generated.

When looking at LA TOFMS data presented in this work, the results might evidence at least some of the structural units identified from Raman scattering spectroscopy data as reported in [20]. For example, observed GeSe_4_^+/−^ clusters confirm the presence of GeSe_4_ tetrahedral entities in the structure of co-sputtered amorphous films. On the other hand, most of the detected species seem not to correspond with local structural units evidenced via Raman spectroscopy. The clusters generated might be formed either due to the deeper fragmentation of the original amorphous structure, or some high mass species that are not considered as part of the original amorphous structure can be formed from the highly energetic ions present in the plasma plume, i.e. by laser ablation synthesis.

#### Selected Ge_x_Se_z_, Bi_y_Se_z,_ and Ge_x_Bi_y_Se_z_ clusters structure optimization

In the past two decades, the atomic and molecular clusters were extensively studied by theoretical chemists to explore their structural and electronic properties. For example, the structures of Ge_*x*_^+/−^, Bi_*y*_^+*/−*^, and Se_*z*_^+/−^ clusters have already been reported^31–35^. In our previous work, we reported on the formation of about 50 and 33 clusters during the laser ablation mass spectrometric study of Ge-Se^27^ and Bi-Se^36^ materials. However, the detailed study of Ge, Bi, and Se binary and ternary combinations clusters’ structure has not yet been reported. In this study we carried out a laser ablation mass spectrometric study of the ternary Ge-Bi-Se system; in this way, many binary Ge_*x*_Se_*z*_^+/−^, Bi_*y*_Se_*z*_^+/−^, and ternary Ge_*x*_Bi_*y*_Se_*z*_^+/−^ clusters are produced and identified. Therefore, the structural properties of selected Ge_*x*_Se_*z*_^+/−^, Bi_*y*_Se_*z*_^+/−^, and Ge_*x*_Bi_*y*_Se_*z*_^+/−^ clusters were investigated via DFT calculations. Because this paper is essentially focused on the mass spectrometric investigation of Ge-Bi-Se materials, only a few simple clusters out of many produced species are selected for the structural optimization. Also, it is well known that the number of possible structural isomers increases drastically with the growing number of atoms in the clusters.

**Figure 6:**
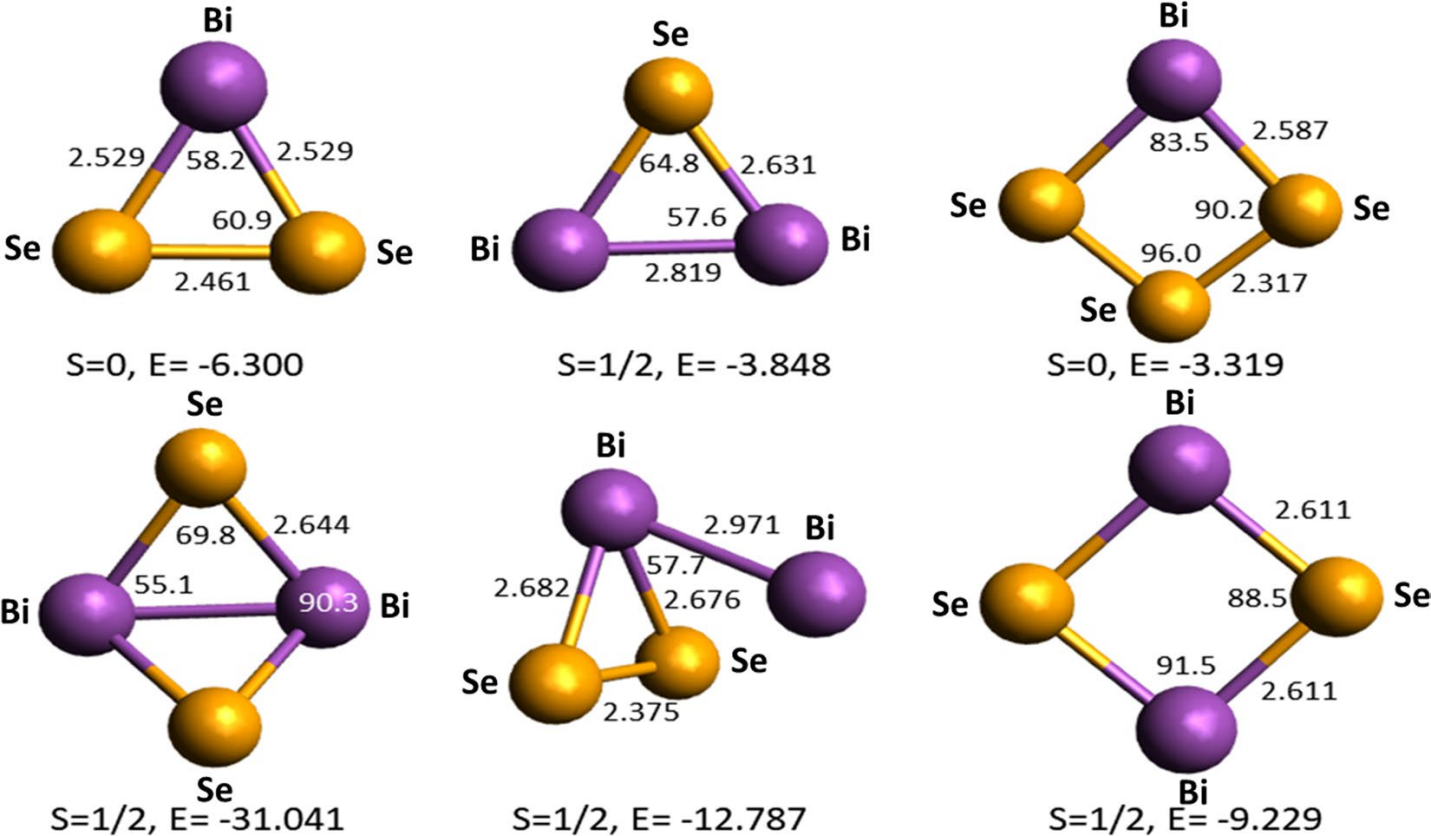
The optimized structures of selected three- and four-atom clusters of the Bi-Se system. The bond length is given in Å, bond angle in degree, energy (E) in kcal/mol, and S stands for the spin. The purple and yellow colored spheres represent bismuth and selenium atoms, respectively.

**Figure 7:**
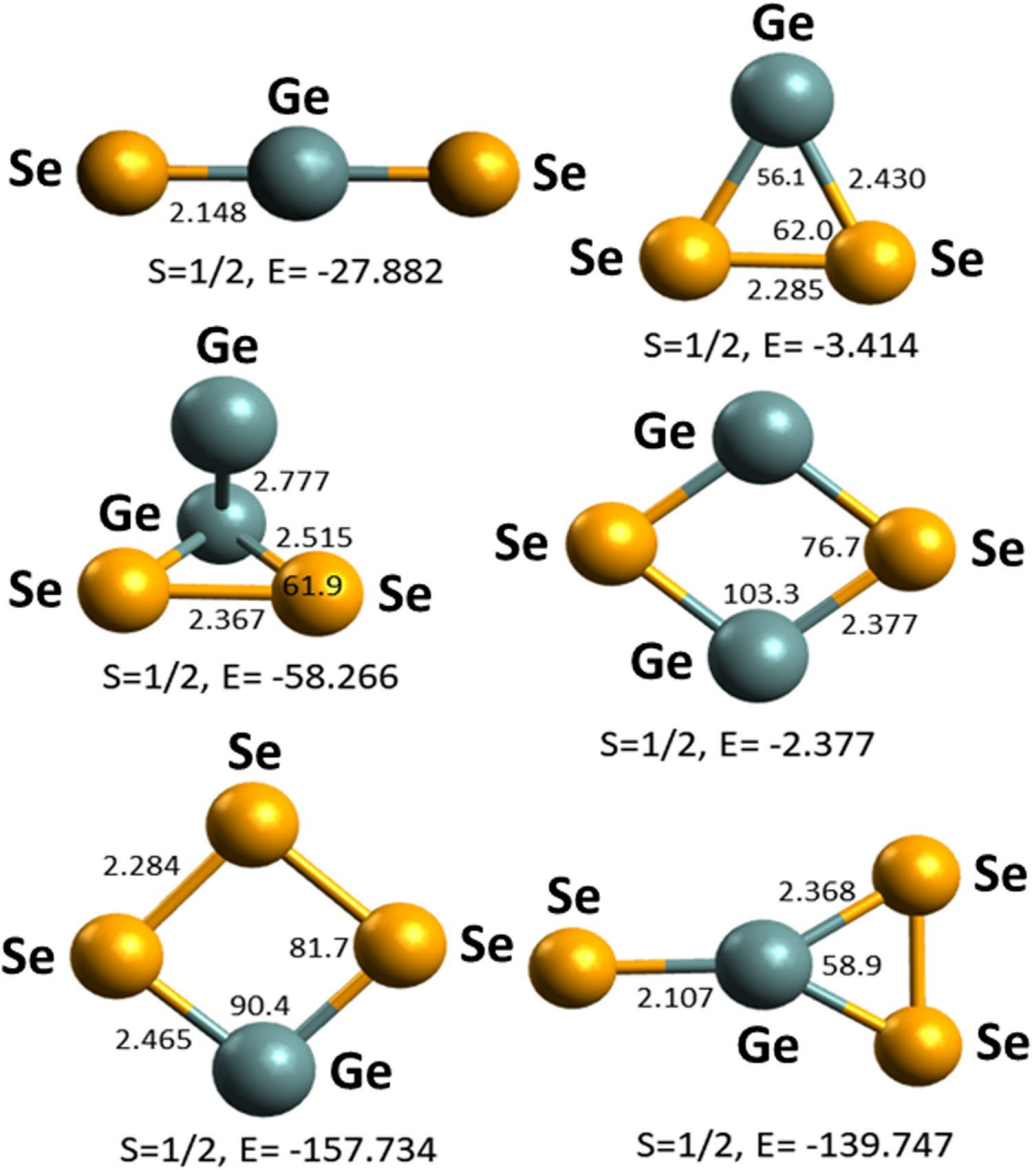
The optimized structures of three- and four-atom clusters of the Ge-Se system. The bond length is given in Å, bond angle in degree, energy (E) in kcal/mol, and S stands for spin. The green and yellow colored spheres represent germanium and selenium atoms, respectively.

**Figure 8:**
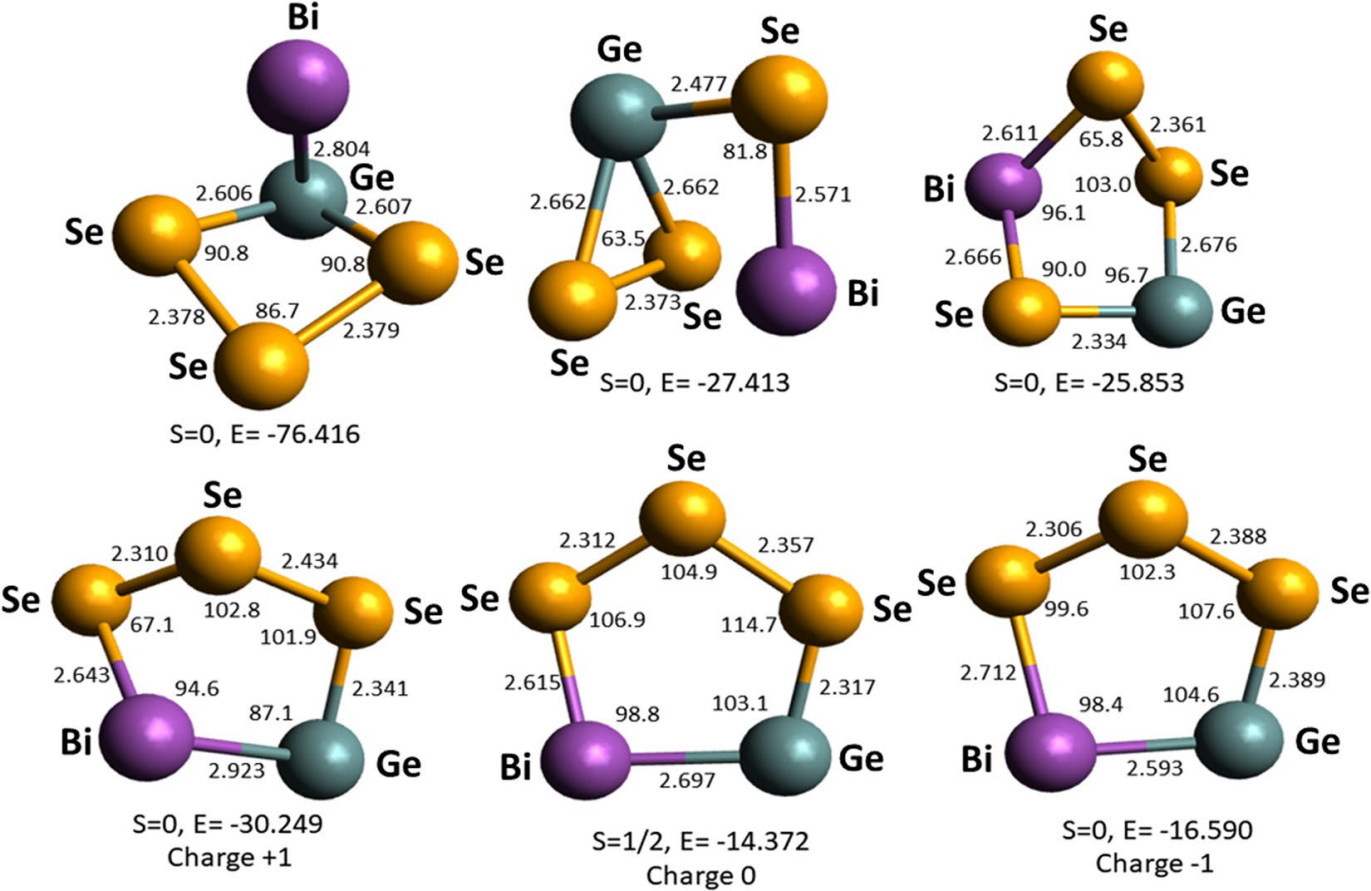
The optimized structural isomers of the GeBiSe_3_ cluster. The last three geometries represent monocationic, neutral, and monoanionic structures. The bond length is given in Å, bond angle in degree, energy (E) in kcal/mol and S stands for spin. The purple, green, and yellow colored spheres represent bismuth, germanium and selenium, respectively.

Different possible isomers of each selected cluster have been studied and the ground state geometries of each cluster with local minima in the potential energy surface (PES) and lowest energy have been determined (Figs. 6–8). The geometry optimization has been performed at the PBE0/TZ2P^37,38^ level of theory using Gaussian software^39^. For an open-shell system with unpaired electrons, an unrestricted approach was used. To ensure that the calculated geometries correspond to the ground state with local minima in the PES, frequencies were computed at the same level of theory. Absence of imaginary frequency confirms that our structures are indeed in the ground state.

The number of the most stable isomers of three- and four-atom binary Bi_*y*_Se_*z*_^+^ clusters (BiSe_2_^+^, Bi_2_Se^+^, BiSe_3_^+^, and Bi_2_Se_2_^+^) are given in Fig. 6. The distance between Bi-Bi and Se-Se near-neighbours is found in the range 2.819–2.971 and 2.317–2.461 Å, respectively, while for the heteroatomic Bi-Se, bond length is 2.529–2.682 Å.

Similarly, the optimization of some selected three- and four-atom Ge_*x*_Se_*z*_^+^ clusters such as GeSe_2_^+^, Ge_2_Se_2_^+^, and GeSe_3_^+^ was performed. The optimized structures are shown in Fig. 7. For each cluster, several isomers were considered for optimization, but only ground state geometries with the lowest energy are shown in Fig. 7. The bond distance between Ge-Ge, Se-Se, and Ge-Se nuclei were found as 2.777, 2.284–2.367, and 2.107–2.515 Å, respectively.

Finally, the GeBiSe_3_ ternary cluster was selected for the structure optimization procedure. The cluster structures were optimized in +1, 0, and −1 charge. Figure 8 shows the optimized structures of the GeBiSe_3_ cluster, the last three geometries represent monocationic, neutral and monoanionic isomers. From the calculated data it was observed that the structural parameters such as bond length, bond angle and the geometry of monocationic, neutral and monoanionic structures do not alter much with the charge. The bond length between two neighbouring nuclei Se-Se, Ge-Se, Bi-Se, and Bi-Ge is in the range 2.306–2.434, 2.317–2.676, 2.571–2.712, and 2.593–2.923 Å, respectively.

## Conclusions

LA with TOFMS analysis of thin films as well as elemental mixtures of Ge-Bi-Se system produces many unary, binary and some ternary clusters. The thin films were prepared via rf magnetron co-sputtering using GeSe_2_ and Bi_2_Se_3_ targets. Six different compositions of thin films were examined, each one producing about 20 different positively and negatively charged clusters. These might be considered as fragments of local structure of studied materials that are present in plasma during thin films deposition. On the other hand, in the case of the elemental mixtures, a higher number (about 28) of clusters were generated at lower laser energy. Finally, structures of some selected binary and ternary clusters were calculated using DFT optimization.

In conclusion, laser ablation time-of-flight mass spectrometry is considered as a useful analytical technique to study amorphous chalcogenide thin films in terms of identification of species present in the plasma phase when the material is exposed to laser pulses. The knowledge of stoichiometry of the species might help to obtain partial structural information of the thin films. Laser ablation of thin films and knowledge of plasma based on TOFMS analysis can also be generally helpful in understanding specific industrial processes such as laser surface treatment and laser additive manufacturing.

## Materials and Methods

### Chemicals

Water used was double distilled from a quartz apparatus Heraeus Quarzschmelze (Hanau, Germany). Germanium, bismuth, selenium (5N purity), acetone, methanol and acetonitrile were purchased from Sigma-Aldrich (Steinheim, Germany). Red phosphorus was bought from Riedel de Haën (Hannover, Germany). Micro-90 (cleaning agent) was from Kratos (Manchester, UK). Xylene (a mixture of isomers) was purchased from Mikrochem Spol. s.r.o. (Pezinok, Slovak Republic) and parafilm from Pechiney Plastic Packaging (Chicago, IL, USA). Silicon wafers used as substrates for the thin films deposition were purchased from ON Semiconductor (Czech Republic). All other reagents were of analytical grade purity.

### Preparation of thin films

Amorphous Ge-Bi-Se thin films were deposited using conventional rf (13.56 MHz) magnetron sputtering technique. The experimental conditions of the deposition process are described elsewhere^23^. For the deposition of the Ge-Bi-Se thin films, commercial polycrystalline GeSe_2_ and Bi_2_Se_3_ sputtering targets (ALB Materials, Inc., Henderson, NV, USA) were used.

### Mixtures of Ge, Bi, and Se

Appropriate amounts of high purity germanium, bismuth, and selenium elements were mixed together in an agate mortar to get the desired composition (1:1:1, 1:1:5, 1:5:1, and 5:1:1 molar ratios). The mixture was then suspended in acetonitrile applying ultrasonication for 1 min and finally used for mass spectrometric measurements.

About 2 mg of each elemental mixture was dispersed separately in parafilm solution prepared by dissolving a piece of (1 cm × 1 cm) parafilm in xylene (2 ml) using ultrasonication, and then used further for the experiments.

### Instrumentation

The surface morphology and chemical composition of the thin films were determined by using a scanning electron microscope (SEM) with an energy dispersive X-ray analyzer (EDS, TESCAN VEGA 3 EasyProbe). The EDS measurements were performed at 3 different spots on the thin films for each sample and averaged. The uncertainty of EDS measurements for the studied films is ±1 at. %. The X-ray diffraction (XRD) technique (D8-Advance diffractometer, Bruker AXS, Germany) was employed to confirm the amorphous state of co-sputtered thin films. The diffraction angles were measured at room temperature from 5 to 65° (2θ) within 0.02° steps.

The target plate was cleaned according to the Shimadzu target cleaning procedure. Specifically, it includes cleaning with water followed by ultrasonic cleaning separately in Micro-90 solution, acetone, and methanol for 15 min and finally rinsing several times with water and acetonitrile.

1 µL of acetonitrile suspension of the sample powder (1 mg/mL) was deposited on the target plate and dried in a stream of air at room temperature and LA TOF mass spectra were recorded. Thin films were fixed on the target plate using adhesive tape; a small portion of the thin films was coated manually with a parafilm solution and then measured.

An AXIMA CFR mass spectrometer from Kratos Analytical Ltd. (Manchester, UK) equipped with a nitrogen laser (337 nm) and a time-of-flight mass analyzer was used to record the mass spectra. The laser repetition rate was set to 5 Hz with a pulse duration of 3 ns. The exposed sample spot size was approximately 150 μm in diameter, giving the laser energy fluence ~1 J cm^−2^.

Mass spectra were recorded in both positive and negative ion modes by accumulating the data from about 1000 laser shots. The laser energy varied in arbitrary units (a. u.) from 0 to 180; this relative scale is used hereafter. External calibration was done using red phosphorus clusters, while the accuracy achieved was below ±50 mDa.

### Software and computation

Theoretical isotopic patterns were calculated using Launchpad software (Kompact version 2.9.3, 2011) from Kratos Analytical Ltd. (Manchester, UK). The calculated structures were visualized using Avogadro: an open-source molecular builder and visualization tool (version 1.2.0., http://avogadro.cc/)^40^.

## Data Availability

The datasets generated and analyzed during the current study are available from the corresponding author on reasonable request.
